# Post-surgery Physical Rehabilitation for Gingivobuccal Sulcus: A Case Report

**DOI:** 10.7759/cureus.72197

**Published:** 2024-10-23

**Authors:** Hrutuja J Karekar, Tejaswini Dafe, Sakshi S Gandole

**Affiliations:** 1 Department of Community Health Physiotherapy, Ravi Nair Physiotherapy College, Datta Meghe Institute of Higher Education and Research, Wardha, IND

**Keywords:** gingivobuccal sulcus, oral cancers, pain management, physical therapy, rehabilitation

## Abstract

The gingivobuccal sulcus, an anatomical term, refers to the area where the gums (gingiva) meet the inside of the cheek (buccal mucosa). It is the groove or fold that forms between the upper or lower gums and the inner surface of the cheek. This area allows movement between the gums and cheeks while talking, eating, or moving the mouth. The gingivobuccal sulcus is a critical anatomical area within the oral cavity that can be affected by various conditions, including oral cancer, leading to significant functional impairment. Surgical intervention in this region, often necessary for tumor resection or trauma repair, can result in challenges related to speech, mastication, and oral hygiene. In order to recover these capabilities and enhance the patient's quality of life, post-surgical rehabilitation is essential. The 43-year-old patient whose main complaints in this article include pain, discomfort, swelling, and trouble chewing had a segmental mandibulectomy during the procedure. Following surgery, physical therapy's primary goals were to relieve pain and restore vital functions. A multidisciplinary strategy is used in this procedure, combining the knowledge of physiotherapists, rehabilitation specialists, and surgeons. The goal was to increase the movement of the jaw, the tongue, and the neck and the swallowing process. The physiotherapy regimens facilitated the patient's recuperation.

## Introduction

Nowadays, cancer is the most prevalent disease affecting humans. Globally, it is estimated that approximately 14.0 million new cases and 8.2 million deaths from cancer occur each year. Oral cavity cancers (OCCs) account for 29.6% of all cancer cases in India. Squamous cell carcinoma (SCC) is a collection of oral cavity malignancies with varying prognoses, symptoms, and routes of dissemination. SCC accounts for 90% of all oral cancers [1,2]. This is due to the fact that common SCC subsites exhibit significant variation based on genetic, cultural, and environmental risk factors. Oral cancer makes up 2%-6% of all cancer cases and 30% of head and neck cancer cases [3]. Of them, the mandibular gingivobuccal (GB) sulcus represents the source of a significant portion of the carcinomas. Surgery is the primary treatment for GB sulcus tumors, which is a subsite of significant anatomical and clinical importance. In India, GB cancer (GBC) is highly prevalent, primarily as a result of tobacco chewing behaviors [4].

In the world, India has one of the highest incidences of oral cancer. In India, mouth cancer ranks third among women and first among men. Eight percent of cancer cases in women and 12% of cases in males are related to oral cancer. Oral cancer in men in India had an age-standardized incidence rate of 10.1 [5]. GBC accounts for approximately 40% of all cases of OCC in Indian communities in Africa and Southeast Asia; in contrast, the risk of GBC in the Western world is approximately 10% [6]. GBC is known as the "Indian oral cancer," quite fittingly. In the Indian subcontinent, GBC is distinct and primarily prevalent in OCC. The overall survival rate of two to five years is about 63% and 53%, respectively, in prestigious institutions. The lower GB complex consists of the buccal mucosa, GB sulcus, lower gingiva, and retromolar trigone [7,8]. Lips in the anterior position; mylohyoid muscle, mandibular alveolar ridge, and teeth in the inferior position; GB regions in the posterior position; circular papillae, tonsillar pillars, and soft palate in the posterior position; and hard palate, maxillary alveolar ridge, and teeth in the superior position form the boundaries of the oral cavity [9,10]. Superior GB complex malignancies are defined as those affecting the buccal sulcus and the upper part of the buccal mucosa [11]. In contrast, those affecting the lower GB complex are classified as retromolar trigone, buccal mucosa, and GB sulcus cancers [12]. In contrast, lower gingivo-oral malignancies, also called "Indian oral cancer," are less aggressive than upper sulcus tumors, even in advanced stages of the disease, and have a better prognosis. Although GB sulcus tumors are easily identified and prevented, they constitute a global health problem [13,14].

In order to minimize problems including trismus, fibrosis, and pain, improve range of motion, and restore oral function after GB sulcus surgery, rehabilitation is essential. To get the best results, physical therapists and dentists frequently need to work together in a multidisciplinary approach. The GB sulcus surgery patient's rehabilitation tactics and results are shown in this case study, underscoring the value of a customized rehabilitation program in promoting healing and improving quality of life.

## Case presentation

Patient information 

A patient at the age of 43 years was experiencing painful non-healing ulcers over the left buccal mucosa, which had persisted for one year. The patient has a history of chewing tobacco for 20 years and smoking for 15 years. Initially, the patient noticed an ulcer that was increasing gradually in size. His pain was intermittent, dull-aching, and non-radiating in nature, which was aggravated by moving his mouth, such as talking and chewing, and relieved with rest. He first visited a hospital in Mumbai for his primary treatment. On May 18, 2024, he had undergone an incisional biopsy. Then, he was referred to our hospital for further treatment. Here, investigation was carried out, which includes contrast-enhanced computed tomography (CECT) of the buccal cavity. Here, he underwent three surgeries. Two on July 13, 2024, which were microvascular reconstruction and neck dissection, and one on July 20, 2024, which was segmental mandibulectomy. After surgery, jaw pain worsened when the mouth was opened or while eating; avoiding demanding mouth movements helped lessen it. The patient was referred to the physiotherapy rehabilitation center as a result of all these complaints. The patient's primary concerns were difficulty speaking, difficulty doing activities of daily living, difficulty chewing and swallowing, and difficulty opening his mouth following surgery.

Clinical findings

Prior to the physical examination on post-operative day four, the patient gave both verbal and written consent. On observation, the patient was supine lying with the head and back elevated to 45°. On inspection, chest wall mobility was observed to be reduced. The use of sternocleidomastoid and scalene muscles (accessory muscles) was seen. On examination, the patient was hemodynamically stable. Breathing was normal and abdomino-thoracic with a respiratory rate of 19 breaths/min. Chest expansion was reduced due to pain on the suture site. On the visual analog scale (VAS) on activity, the score was eight, and on the rest, the score was four before rehabilitation. On motor examination, the range of motion of the temporomandibular joint and cervical joint was decreased.

Diagnostic findings

**Figure 1:**
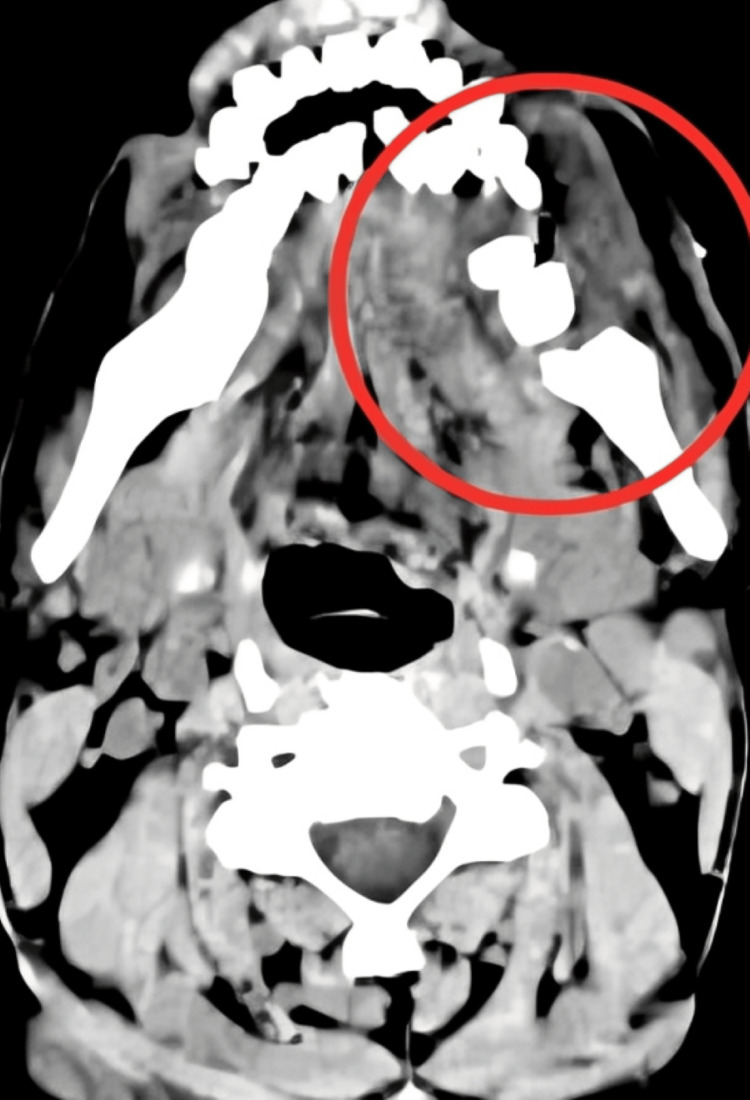
CECT after segmental mandibulectomy CECT: contrast-enhanced computed tomography

Figure 1 shows CECT performed after segmental mandibulectomy; a part of the mandible is resected. The extent and size of the original mass lesion were determined through a CECT. A heterogeneous lobulated mass at the buccal space on the right aspect was seen. There was no apparent fat plane connecting this mass to the right masseter muscle, and it continued posteriorly to the left masticator area. 

Physiotherapy intervention

The patient was educated and counseled about the importance of physiotherapy post-operatively before starting the treatment. Interventions are given below in Table 1.

**Table 1:** Intervention

Problem list	Intervention	Dosage	Rationale
1. Limited mouth opening	TheraBite is a hand-held device used to stretch the jaw; Rocabado exercises	TheraBite for five sessions once a day (30-sec stretch for each opening); Rocabado exercises for six exercises for six times	To improve jaw range of motion, reduce stiffness, and prevent trismus, which can be a complication of the condition
2. Limited jaw mobility	Jaw mobility exercises-manual stretching, jaw opening against resistance, mouth opening and closing exercises, mirror therapy	10 repetitions, twice a day	To improve jaw mobility
3. Swelling and inflammation in the gingivobuccal area	Application of cold compresses	10-15 minutes per session	To reduce inflammation and control swelling in the affected area
4. Pain during mouth movements	Pain management techniques include gentle massage, positioning, postural re-education, and relaxation		To decrease pain, facilitate participation in exercises, and improve overall comfort
5. Difficulty in chewing and swallowing	Orofacial exercises, strengthening, Mendelsohn maneuver	Exercises for 10 repetitions, two times a day; Mendelsohn maneuvers for 10 to 12 times once a session	Enhance muscle strength by improving the ability to chew and swallow effectively
6. Impaired speech due to limited tongue mobility	Tongue exercises (circular motion, diagonal, up and down movement of tongue), tongue stretches, resistance exercises, reading a book or newspaper aloud	10 repetitions, two times a day	To improve tongue mobility, which is essential for articulation and speech clarity
7. Reduced functional capacity of lungs	Inspiratory muscle training, thoracic expansion, diaphragmatic breathing	10 repetitions, three times a day (with breath-hold for 2-3 sec)	To improve and maintain the functional capacity of the lungs
8. Reduced cervical range of motion	Chin tucks; active range of motion exercises of cervical flexion, extension, lateral flexion, and side flexion; neck stretch	10 repetitions, twice a day	To improve and maintain cervical range of motion
9. Poor oral hygiene due to difficulty in maintaining	Education on proper oral hygiene practices was given, such as avoidance of brushing over surgical sites, use of a soft-bristled toothbrush, usage of a proper medicated mouthwash, and avoidance of stretching cheeks	Twice a day (morning and night)	To prevent further infection, promote healing, and maintain overall oral health
10. Decreased flexibility of collagen fibers	Ultrasound therapy	3 MHz for seven minutes, once a day	To hasten recuperation, enhance the flexibility of collagen fibers, and alleviate pain
11. Reduction in strength of fibrous tissues and immobilized scar	Scar mobilization, along with kneading and facial massage	Once a day post-suture removal	To enhance the strength of underlying fibrous tissues, mobilize scars
12. Anxiety and stress	Relaxation techniques: deep breathing, guided imagery visualization		To reduce anxiety and stress, which can exacerbate pain and interfere with recovery

Follow-up and outcome measure

A follow-up evaluation was carried out after a therapeutic intervention. VAS shows that the activity score was six and the rest score was two after rehabilitation (Table 2), the cervical range of motion was increased after rehabilitation (Table 3), and the range of motion of the temporomandibular joint was increased after rehabilitation (Table 4). Oral Health Impact Profile (OHIP-14) (Table 5) [15] shows a score of 19 (mild to moderate impact) before rehabilitation, and after rehabilitation, it shows a score of 42 (high impact). These were among the outcome measures that were collected both before and after rehabilitation. Following rehabilitation, it was discovered that the outcome measure scores had increased.

**Table 2:** Visual analog scale (VAS)

	Pre-rehabilitation	Post-rehabilitation
On activity	8/10	6/10
On rest	4/10	2/10

**Table 3:** Cervical range of motion

	Pre-rehabilitation	Pre-rehabilitation	Post-rehabilitation	Post-rehabilitation
	Left	Right	Left	Right
Lateral flexion	0-12°	0-12°	0-30°	0-40°
Rotation	0-40°	0-50°	0-70°	0-80°
Flexion	0-50°	0-50°	0-80°	0-80°
Extension	0-30°	0-30°	0-50°	0-50°

**Table 4:** Range of motion of temporomandibular joint (TMJ)

	Pre-rehabilitation	Post-rehabilitation
Opening of mouth	12 mm	35 mm
Lateral movement	4 mm	7 mm
Protrusion	2 mm	6 mm

**Table 5:** Oral Health Impact Profile (OHIP-14)

	Pre-rehabilitation	Post-rehabilitation
Oral Health Impact Profile (OHIP-14)	19/56	42/56

## Discussion

Post-surgical rehabilitation in patients with GB sulcus carcinoma is challenging due to the extensive nature of the surgery, which often involves critical oral and facial structures. These procedures can result in significant functional impairments, including trismus (restricted mouth opening), dysphagia (difficulty swallowing), and facial asymmetry, all of which can severely affect the patient's quality of life. Effective rehabilitation requires a multidisciplinary approach involving physical therapists, speech therapists, and psychologists, among others. Early intervention with a structured rehabilitation program is essential to prevent long-term complications and promote functional recovery [16,17].

According to studies, stretching exercises performed with devices such as TheraBite or wooden spatulas, which are fundamentally used to increase mouth opening, should be prescribed by a physiotherapist. The physiotherapist should also educate the patient regarding how these exercises can prevent further limitations in the opening and closing of the mouth. According to Kamstra et al., mouth openness expanded by an average of 5.4 mm following TheraBite workouts. The findings show that, after controlling for the impact of the medical center, there is a decreased likelihood of a 5 mm or greater increase in mouth opening if the interval between the commencement of workouts and cancer treatment is prolonged [18]. According to McCullough and Kim, the data from this study lend credence to the idea of using the Mendelsohn maneuver as a swallow physiology exercise. After therapy, there were notable improvements in the correlations between the bolus flow, upper esophageal sphincter (UES) opening, and hyoid movement measurements. There were improvements noted [19]. In the case study by Mangulkar et al., an older patient with lip SCC underwent a mandibulectomy; as a result, he developed severe trismus and was unable to speak or drink water. After a course of physiotherapy, including Rocabado exercises, the patient made substantial progress and restored jaw-opening movement. Patients who experience symptoms related to the head and neck, such as trismus after a mandibulectomy, should regularly perform jaw stretching exercises, orofacial strengthening, and tongue exercises, which will help in managing the symptoms [20].

In this case, the patient's structured rehabilitation program, initiated immediately after surgery and tailored to address specific complications, led to significant functional recovery [21]. The multidisciplinary approach-combining physical therapy, speech therapy, and psychological support-was essential in addressing the diverse needs of the patient. This holistic strategy aligns with the recommendations in the literature for managing complex cases of head and neck cancer, where the goal is not only to improve physical function but also to enhance the overall quality of life [22].

## Conclusions

This case report demonstrates the critical role of physical rehabilitation in the recovery of patients after surgery for GB sulcus carcinoma. A comprehensive, patient-specific rehabilitation plan can lead to substantial improvements in function and quality of life, emphasizing the need for early and sustained intervention.
